# CoCiter: An Efficient Tool to Infer Gene Function by Assessing the Significance of Literature Co-Citation

**DOI:** 10.1371/journal.pone.0074074

**Published:** 2013-09-23

**Authors:** Nan Qiao, Yi Huang, Hammad Naveed, Christopher D. Green, Jing-Dong J. Han

**Affiliations:** 1 Chinese Academy of Sciences Key Laboratory of Computational Biology, Chinese Academy of Sciences-Max Planck Partner Institute for Computational Biology, Shanghai Institutes for Biological Sciences, Chinese Academy of Sciences, Shanghai, China; 2 Center of Molecular Systems Biology, Institute of Genetics and Developmental Biology, Chinese Academy of Sciences, Beijing, China; 3 University of Chinese Academy of Sciences, Beijing, China; King Abdullah University of Science and Technology, Saudi Arabia

## Abstract

A routine approach to inferring functions for a gene set is by using function enrichment analysis based on GO, KEGG or other curated terms and pathways. However, such analysis requires the existence of overlapping genes between the query gene set and those annotated by GO/KEGG. Furthermore, GO/KEGG databases only maintain a very restricted vocabulary. Here, we have developed a tool called “CoCiter” based on literature co-citations to address the limitations in conventional function enrichment analysis. Co-citation analysis is widely used in ranking articles and predicting protein-protein interactions (PPIs). Our algorithm can further assess the co-citation significance of a gene set with any other user-defined gene sets, or with free terms. We show that compared with the traditional approaches, CoCiter is a more accurate and flexible function enrichment analysis method. CoCiter is freely available at www.picb.ac.cn/hanlab/cociter/.

## Introduction

A basic task in biological research is to uncover or validate the functions of genes, such as candidate genes from a genetic screen and differentially expressed genes from microarray or RNA-seq experiments. A quick way of inferring functions is by using the gene function enrichment analysis tools, such as DAVID [1] and BiNGO [2], which infers overrepresented functions in a gene set from Gene Ontology (GO) [3] or Kyoto Encyclopedia of Genes and Genomes (KEGG) [4] curated terms and pathways. However, the drawback of GO/KEGG-related functional association analyses is that both GO and KEGG only maintain a controlled vocabulary of terms, which prevents them from analyzing genes that do not have GO/KEGG annotations [5].

PubMed is the largest biomedical knowledgebase that is comprised of over 21 million abstracts and is growing at an alarming rate. The PubMed abstracts contain all the essential information of the papers and therefore are an important resource for text mining. In addition to ranking articles [6]–[9] or predicting Protein-Protein Interactions (PPIs) [10], [11], scientific literatures are also be widely used to interpret the functions of a gene set [12]–[16].

Here, we have developed an application program called “CoCiter” that is able to evaluate the significance of co-citation for any gene set from the 8,077,952 genes in the National Center for Biotechnology Information (NCBI) Entrez gene database [17], by using a text mining approach against the up-to-date Medical Literature Analysis and Retrieval System Online (MEDLINE) literature database. CoCiter can evaluate the significance of co-citation for two types of queries: 1) query gene set with any pre-defined/manually-curated gene set, e.g. gene sets from GO/KEGG, 2) query gene set with any user-defined free term set, e.g. “diabetes” or “leukemia”. We demonstrate that CoCiter provides a flexible and more precise approach to analyzing gene set functions, compared with the traditional function enrichment analysis.

## Materials and Methods

### Citation for a gene

HomoloGenes for *Homo sapiens*, *Mus musculus*, *Drosophila melanogaster* and *Caenorhabditis elegans* were downloaded from NCBI FTP site in October 2011.

To find the co-citation literature related to a gene, Caipirini [18] and Martini [15] use the “gene2pubmed” dataset (a manually curated gene to PubMed literature relationship dataset provided by NCBI); CoPub [19] uses regular expression to search against Medline abstracts. Although the “gene2pubmed” dataset contains manually curated information for gene co-citation, its coverage is small – most genes have less than 10 co-citations (Figure S1). Additionally, using full text or regular expressions to search for the gene symbol will result in a large number of reports, most of them being false positives and unrelated to the gene. CoCiter checks against both the NCBI “gene2pubmed” dataset (downloaded from NCBI FTP in Oct, 2011) and an expanded “gene2pubmed” dataset based on our own mapping. The expanded dataset is generated by using the NCBI E-utilities [20] to search for PubMed abstracts that contain an Entrez gene name and the word “gene”, e.g. “AKT1 gene”, to assure the accuracy of the query. We manually examined this rule on 50 randomly selected human genes (Table S1 in File S1). If a query finds too many reports, the top 500 best-matched records are retrieved (in the original “gene2pubmed” dataset, only 0.007% of total genes have >500 co-citations each). The original gene2pubmed dataset contains 7,565,397 records. With this expansion, the dataset now contains 38,261,321 records.

### Citation for a term in the form of free text

To find the co-citations related with a free text term, we use Apache Lucene v3.4.0 (a high-performance, open source full text search engine, http://lucene.apache.org) to search the PubMed abstracts for the given term. To satisfy both the accuracy and speed of the full text search, Lucene has indexed all the 1,640,530 abstracts (downloaded from NCBI on Oct. 2011) of 16 common organisms as listed in Table S2 in File S1. For a single word, Lucene finds its related abstracts by scanning the index. For a phrase, Lucene first breaks it into single words for index scanning, and then it finds complete matches while allowing no more than one insertion between the words. We used Lucene's “NIOFSDirectory” function to store the index file in the file system; the “StandardAnalyzer” function to analyze the full text; and the “IndexSearcher” function to search a free phrase against the stored index.

### Assessing the significance of co-citation

We define a log-transformed paper count, which we call co-citation impact (CI), to penalize study biases for star-like genes or terms. CI of a gene/term with another gene/term is defined as 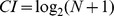, where *N* is number of abstracts both gene/term appears. CI of a gene/term set *A* with another gene/term set *B* is defined as 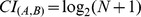, where 
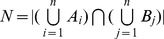
, where *A_i_* is the set of PubMed abstracts within which the *i*-th gene/term in set *A* is cited, and *B_j_* is the set of abstracts within which the *j*-th gene/term in set *B* is cited. Thus, *N* represents the total counts of abstracts that have at least one gene/term in set *A* and one gene/term in set *B* co-cited.

To assess the significance of co-citation of gene set *A* with another gene set or term set *B*, a Monte Carlo approach is used to evaluate random expectations. CoCiter randomly selects 1000 gene sets with the same size as *A* within the same species, and the *CI_(random, B)_* is calculated for each of the 1000 random gene sets with *B*. The permutation p value is then defined as the number of times that 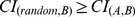 divided by 1000.

### Datasets

Three datasets are used to evaluate the performance of CoCiter against other tools: The first dataset is a small dataset that has been used by Martini [15]. It contains 269 *Arabidopsis* genes associated with disease resistance mechanisms and 514 randomly selected genes with no clear evidence relating them to any disease (Table S3 in File S2). The second dataset has been used by Rhodes *et al.* [21] as a gold standard negative set of protein interactions. It contains 1397 human genes that encode plasma membrane proteins and 2224 human genes that encode nuclear proteins (Table S4 in File S2). The third dataset is a large dataset that we manually curated (see Supplemental Methods in File S1). It contains 2097 pairs of gene sets as gold standard positive (GSP) (Table S5 in File S2) and 603 pairs of gene sets as gold standard negative (GSN) (Table S6 in File S2).

## Results

### Overview

**Figure 1:**
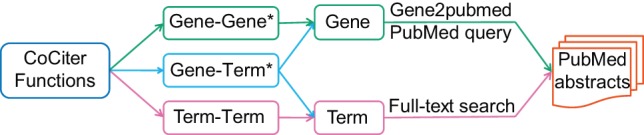
Schematic view of the functions in CoCiter. The Gene-Gene and Gene-Term association analysis functions (asterisked) include significance test for the result.

CoCiter mainly includes three functions: CoCiter Gene-Gene association analysis calculates the co-citation significance between two gene sets; CoCiter Gene-Term association analysis calculates the co-citation significance between one gene set and a term set; CoCiter Term-Term co-citation analysis only provides the co-citation information between two term sets, as it is impossible to evaluate the background distribution of the unlimited number of free terms (Figure 1).

In the scientific literature, the study bias for different species is huge. For example, the number of reports regarding the four model organisms human, mouse, fly and worm vastly outnumber most other species. To take advantage of the large literature base of the four model organisms, CoCiter combines the information from homolog genes for human, mouse, fly and worm for any query gene in a co-citation search. This makes the searches effective even for a gene that is poorly studied in some species.

### CoCiter Gene-Gene and Gene-Term association analysis functions

CoCiter Gene-Gene association analysis function takes two gene sets as input. The first gene set is regarded as a query gene set with unknown functions, and the second gene set is regarded as a target gene set with known functions, such as a gene set from an annotation database or a manually curated one. CoCiter first finds the co-citation PubMed abstracts for the two gene sets, and then uses co-citation impact (CI) of one gene set (query) with another gene set (target) as the metric to measure the level of co-citation (MATERIALS AND METHODS).

The CoCiter Gene-Term association analysis function takes one gene set and one term set as input. The gene set is regarded as a query gene set with unknown functions, and the term set contains free terms (gene functions, cell components, diseases, etc.). To determine if the gene set is significantly co-cited with the terms in the term set, CoCiter first finds the PubMed abstracts co-cited for the gene set and the term set, the latter of which uses full text searches for each term against the PubMed abstracts (MATERIALS AND METHODS). The CI of the gene set with the term set is calculated to measure the level of co-citation (MATERIALS AND METHODS).

The query results include CI, significance of CI based on 1000 permutation tests, the co-cited PubMed papers, and their hyperlinks to the PubMed database. To avoid misrepresentation of CI by star papers, we also provide background-adjusted CI, which is defined as *CI(A,B) – average CI(random, B)*. The “one-to-all” result section lists the co-citation statistics of each gene in one gene set with all the genes in another gene set, ranked by CIs. The co-citation abstracts are shown with query genes/terms highlighted to facilitate visual examination of the results. The star papers are listed separately at the bottom of the paper list (Supplemental Notes in File S1, Table S7 in File S2).

### CoCiter web application

The CoCiter web application is well optimized for speed and performance. First, we pre-indexed the citations for all the Entrez genes, and stored them in a MySQL database to facilitate fast retrieval. Second, we pre-indexed all the titles and abstracts to accelerate term co-citation by taking advantage of the functionalities in Lucene search engine for free text search (see MATERIALS AND METHODS). Third, after generating the permutated gene sets, we made a tradeoff of speed at the expense of space – rather than searching the MySQL database numerous times, we retrieved the co-citation information once from MySQL database for all the genes in the permutation sets, and stored this information into memory for downstream computation, which greatly reduced MySQL accession. A 1000×1000 Gene-Gene query with 1000 permutations takes <4 minutes. The results can be viewed online or downloaded as a zip file. In addition to graphical user interfaces with detailed tutorials, CoCiter also provides a Simple Object Access Protocol (SOAP) application programming interface (API) for more sophisticated users to test against GO/KEGG or user-defined gene sets and term sets. A Python script is provided to facilitate customization. A larger number of permutations with Benjamini-Hochberg correction for multiple testing [22] can be set as an optional parameter through the API. The datasets for the web application will be updated semi-annually.

### Comparison of CoCiter with other co-citation tools

Other gene function analysis tools mainly fall into two categories: GO-based and text mining-based. Brief introductions and comparisons of these tools with CoCiter are shown in Table 1. A major difference between the GO-based and text mining-based tools is that the text mining-based tools are able to accept user-defined terms as input for functional association analysis. We first compared CoCiter with three representative programs from either category, FatiGO (first category), Martini (second category) and Marmite [16] (second category), using one small dataset and one medium dataset. Lastly, we illustrated the high sensitivity and specificity of CoCiter using ROC curves based on a large dataset.

**Table 1:** Unique features of CoCiter compared with those of existing gene function analysis tools.

Category	Name	Input	Species	Type	Gene function enrichment analysis	Compare to user defined gene sets	Compare to user defined term sets
**GO-based**	**GOFFA**	Genes	3	ArrayTrack plug-in	√		
	**BinGO**	Genes	24	Cytoscape plug-in	√		
	**ClueGO**	Genes	14	Cytoscape plug-in	√		
	**ProfCom**	Genes	6	Web + API	√		
	**DAVID**	Genes	Many	Web + API	√		
	**FatiGO+**	Genes	10	Web	√	√	
**Text mining -based**	**iHOP**	Genes	8	Web			
	**Caipirini**	Genes and terms	Many	Web + API			
	**MEDIE**	Genes and terms	Many	Web			
	**Info-pubmed**	Genes	Many	Web			
	**GeneWizard**	Genes and terms	Many	Standalone	√^1^		
	**CoPub**	Genes and terms	3	Web + API	√^1^		
	**Marmite**	Genes	Many	Web	√^2^	√	
	**Martini**	Genes and terms	Many	Web + API	√^3^	√^3^	√^3^
	**CoCiter**	Genes and terms	Many	Web + API	√	√	√

1 by utilizing GO enrichment analysis.

2 limited to a small set of predefined terms.

3 only finds the differences between two conditions (conditions could be genes or terms) based on key words.

The first dataset (MATERIALS AND METHODS) is a small dataset containing 269 disease resistance genes and 514 randomly selected genes. Given this dataset, three questions were asked: 1) Are these disease resistance genes related with the term “disease resistance”? 2) Are these randomly selected genes related with the term “disease resistance”? 3) Are these disease resistance genes related with these randomly selected genes?

CoCiter answered all of these questions correctly. Through the CoCiter Gene-Term function, CoCiter found that these disease resistance genes were significantly co-cited with the term “disease resistance” (p<0.001), and the randomly selected genes were not significantly related with the term “disease resistance” (p = 0.337). Through the CoCiter Gene-Gene function, CoCiter found that the disease resistance genes were not significantly co-cited with the randomly selected genes (p = 0.979) (Table 2).

**Table 2:** Performances of CoCiter, FatiGO, Martini and Marmite on the disease resistant and randomly selected dataset.

Tools	Comparison	Time	P	CI	Description
CoCiter	Disease resistance gene vs. Disease resistance term	4 sec	<0.001	6.3576	
	Random genes vs. Disease resistance terms	7 sec	0.337	4.1699	
	Disease resistance genes vs. random genes	9 sec	0.979	10.3859	
FatiGO	Disease resistance gene vs. Disease resistance term background	4 min	<0.001	defense response, immune response	Unable to accept user defined terms
	Random genes vs. Disease resistance term background	4 min	NA	Nothing enriched in these genes	Unable to accept user defined terms
	Disease resistance gene vs. randomly picked gene	2 min	<0.001	response to stress, defense response, immune response	
Martini	Disease resistance gene vs. Disease resistance term	NA	NA	NA	too many entries to carry on
	Random selected genes vs. Disease resistance terms	NA	NA	NA	too many entries to carry on
	Disease resistance genes vs. random genes	<30s	<0.001	disease, resistance, avirulent, pathogen, plant diseases	
Marmite	Disease resistance gene vs. Disease resistance term	NA	NA	NA	Unable to accept user defined terms
	Random gene vs. Disease resistance term	NA	NA	NA	Unable to accept user defined terms
	Disease resistance gene vs. randomly picked gene	<30 sec	NA	NA	No entities found

* Strike-through fonts indicate unavailable functions.

In comparison, FatiGO was unable to compare the gene set with free terms. Using GO terms to compare the disease resistance genes with background, FatiGO found some disease resistance GO terms, such as “defense response” and “immune response”, and it found nothing for the randomly selected genes. Comparing the disease resistance genes with the randomly selected genes, FatiGO found some disease resistance GO terms, such as “response to stress”, “defense response” and “immune response”. Martini failed to compare the gene set with the term “disease resistance” because it was unable to handle the large number of abstracts returned from the queries, but it found that the keywords “disease resistance”, “virulent”, “pathogen” and “plant diseases” are significantly enriched in the abstracts containing the disease resistance genes compared with those for the random genes. Therefore, as listed in Table 1, although Martini can compare two gene sets by finding the enriched keywords in one gene set compared with the other, it cannot identify functional similarities between the gene sets, and its inability to handle large numbers of abstracts precludes its practical use and comparison to CoCiter. Marmite cannot compare a gene set with user defined terms. It only compares two gene sets to see whether a small number of predefined terms, such as those related to diseases, are significantly enriched in one gene set when using the other gene set as background. When comparing the disease resistance genes with the randomly selected genes, it found no significantly enriched predefined terms (Table 2).

The second dataset (MATERIALS AND METHODS) is a medium-scale dataset that contains 1397 genes encoding plasma membrane proteins and 2224 genes encoding nuclear proteins. Given this dataset, the following three questions can also be asked: 1) Are these plasma membrane genes related to the term “plasma membrane”? 2) Are these nuclear genes related to the term “nucleus”? 3) Are these plasma membrane protein-encoding genes related to these nuclear protein-encoding genes?

CoCiter again answered all of these questions correctly. Through the CoCiter Gene-Term function, CoCiter found that the plasma membrane genes were significantly co-cited with the term “plasma membrane” (p<0.001), and the nuclear genes were significantly related with the term “nucleus” (p<0.001). Through the CoCiter Gene-Gene function, CoCiter found that the plasma membrane genes were not significantly co-cited with the randomly selected genes (p = 0.072) (Table 3).

**Table 3:** Performances of CoCiter, FatiGO, Martini and Marmite on the nuclear and plasma membrane protein-coding dataset.

Tools	Comparison	Time	P	CI	Description
CoCiter	Plasma membrane protein-coding genes vs. plasma membrane term	1 min 15 sec	<0.001	12.3923	
	Nuclear protein coding genes vs. nucleus term	1 min 50 sec	<0.001	12.9814	
	Plasma membrane protein coding genes vs. nuclear protein-coding genes	5 min 15 sec	0.072	14.4227	
FatiGO	Plasma membrane protein-coding genes vs. plasma membrane term background	18 min	<0.001	membrane fraction, extrinsic to plasma membrane, lateral plasma membrane	Unable to accept user defined terms
	Nuclear protein-coding genes vs. nucleus term backgroundz	18 min	<0.01	chromosome, ribonucleoprotein complex, pronucleus, membrane fraction, endomembrane system, extracellular space	Unable to accept user defined terms
	Plasma membrane protein coding genes vs. nuclear protein coding genes	13 min	<0.001	extracellular matrix, extracellular region part, extracellular space, basolateral plasma membrane, lateral plasma membrane, extrinsic to plasma membrane, Golgi apparatus, organelle membrane, endomembrane system, nuclear part, chromosome	
Martini	Plasma membrane protein-coding genes vs. plasma membrane term	NA	NA	NA	Too many entries to carry on
	Nuclear protein coding genes vs. nucleus term	NA	NA	NA	Too many entries to carry on
	Plasma membrane protein coding genes vs. nuclear protein-coding genes	1 day 8 hour	<0.001	transmembrane, membrane, plasma membrane, subnucleus, nucleus	
Marmite	Plasma membrane protein-coding genes vs. plasma membrane term	NA	NA	NA	Unable to accept user defined terms
	Nuclear protein coding genes vs. nucleus term	NA	NA	NA	Unable to accept user defined terms
	Plasma membrane protein coding genes vs. nuclear protein-coding genes	<1 min	0.057	Cancer	

* Strike-through fonts indicate unavailable functions.

In comparison, FatiGO was unable to compare the gene set with terms. Using GO terms to compare the plasma membrane or nuclear protein-encoding genes with background, FatiGO found some plasma membrane or nucleus-related GO terms, respectively (Table 3). However, when comparing the two gene sets, FatiGO found many GO terms, but not all of them are related to plasma membrane or nucleus, they instead include many other “false positive” compartments, such as “extracellular matrix”, “Golgi apparatus”, “organelle membrane”, “endomembrane system”, “chromosome”, etc. (Table 3). Martini failed to compare the respective gene sets with the terms “plasma membrane” and “nucleus” because it was unable to handle the large number of abstracts returned by the query, but it found some plasma membrane and nucleus related keywords enriched in the abstracts containing the plasma membrane protein coding genes when using nuclear protein coding genes as background (Table 3). Marmite was unable to compare the gene sets with terms. Using the nuclear genes as background, Marmite only found the disease term “cancer” from the plasma membrane gene set, which was not significantly enriched over background (p = 0.057) (Table 3).

We further manually curated a third dataset, which contains 2176 pairs of gene sets with similar functions as GSP and 603 pairs of gene sets with distinct functions as GSN (MATERIALS AND METHODS). Such big datasets can be easily handled by CoCiter, but are great challenges to the other tools because CoCiter computes much faster compared with the other tools (Table 2 and 3) and provides an SOAP API to facilitate the customized programming.

### Comparison of CoCiter with GO/KEGG-based functional enrichment analysis

Previous literature co-citation analysis cannot assess the statistical significance of the co-citation, while CoCiter can. This gives CoCiter the unique ability of assessing functional enrichment of a gene set as otherwise conventionally done with GO/KEGG-based functional enrichment tests. The question is: how does CoCiter perform in these tasks compared to conventional analysis? We therefore used the third dataset described above to compare the sensitivity and specificity of CoCiter with the conventional GO/KEGG-based functional enrichment analysis.

**Figure 2:**
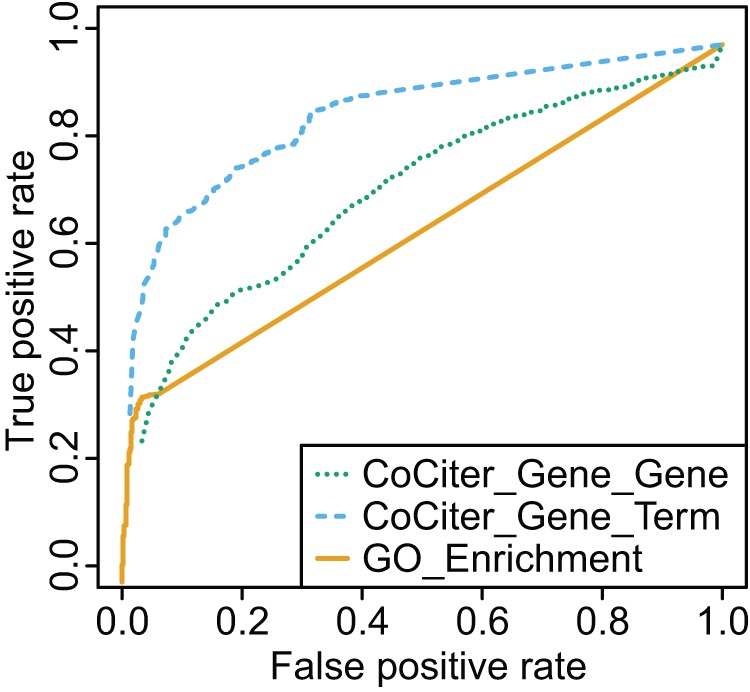
ROC curves of CoCiter and GO enrichment analysis by Fisher exact test. The analysis was based on 2097 gold standard positives (GSP) and 603 gold standard negatives (GSN) selected from the overlapping and non-overlapping GO and KEGG annotations, respectively (Supplemental Methods in File S1). The curve for CoCiter_Gene_Gene association function was obtained by using the KEGG genes and GO genes as input, while that for CoCiter_Gene_Term association function was obtained using the KEGG pathway keywords as terms and GO genes as input.

Using these GSP and GSN datasets, we plotted the Receiver Operator Characteristic (ROC) curves for three different methods – CoCiter Gene-Gene, Gene-Term association and a routine GO enrichment analysis represented by “Fisher's exact test” between gene sets [23]. Judging by the area under the curve (AUC), the CoCiter Gene-Gene association analysis (AUC  = 0.75) is slightly better than the “Fisher's exact test” method (AUC  = 0.65). However, the performance of CoCiter Gene-Term association analysis shows a remarkable improvement (AUC  = 0.88) over the other two methods (Figure 2).

As case studies, we illustrated the advantage of using CoCiter by examining the significance of association to gene sets or term sets on all or part of the GSP and GSN gene and term sets detected by CoCiter or the GO-based analysis (Table 4, Table S8 in File S2). For example, among the 20 representative comparisons at Fisher exact tests with border line significance (P = 0.01) for the GO term enrichment analysis, when the GSP gene set in the KEGG “hsa00071: Fatty acid metabolism” pathway was tested against the GO term “GO:0000038∼very-long-chain fatty acid metabolic process”, “hsa04310: Wnt signaling pathway” against “GO: 0017147∼ Wnt-protein binding” or “hsa04070: Phosphatidylinositol signaling system” against “GO: 0008526∼ phosphatidylinositol transporter activity”, they were tested as insignificant and are hence false negatives, but they were tested as significant by the CoCiter gene-gene and gene-term analyses. When the GSN gene set in the “hsa05040: Huntington's disease” pathway was tested against “GO:0030685∼ nucleolar preribosome”, “hsa00561: Glycerolipid metabolism” against “GO:0010033∼ response to organic substance” or “hsa04614: Renin-angiotensin system VS. GO:0004245∼ neprilysin activity”, they were tested as significant and are hence false positives, but they were tested as insignificant by the CoCiter gene-gene and gene-term analyses.

**Table 4:** Significance of association for 10 GSP and GSN gene and term sets detected by CoCiter or the GO-based analysis.

ID	Type	CoCiter gene- gene analysis	CoCiter gene- term analysis	Fisher's exact test
hsa00071: Fatty acid metabolism VS. GO:0000038∼ very-long-chain fatty acid metabolic process	GSP	0	0.002	0.010020764
hsa04310: Wnt signaling pathway VS. GO:0017147∼ Wnt-protein binding	GSP	0	0	0.010598356
hsa04070: Phosphatidylinositol signaling system VS. GO:0008526∼ phosphatidylinositol transporter activity	GSP	0.004	0.003	0.011084552
hsa00252: Alanine and aspartate metabolism VS. GO:0009067∼ aspartate family amino acid biosynthetic process	GSP	0.014	0.006	0.011268153
hsa04540: Gap junction VS. GO:0005243∼ gap junction channel activity	GSP	0.002	0	0.011605198
hsa04010: MAPK signaling pathway VS. GO:0043409∼ negative regulation of MAPKKK cascade	GSP	0	0.006	0.012145396
hsa04020: Calcium signaling pathway VS. GO:0051925∼ regulation of calcium ion transport via voltage-gated calcium channel	GSP	0	0.008	0.012581597
hsa04910: Insulin signaling pathway VS. GO:0032868∼ response to insulin stimulus	GSP	0.004	0.004	0.013070018
hsa04210: Apoptosis VS. GO:0042771∼ DNA damage response, signal transduction by p53 class mediator resulting in induction of apoptosis	GSP	0	0	0.013908051
hsa04510: Focal adhesion VS. GO:0051895∼ negative regulation of focal adhesion formation	GSP	0	0	0.014279408
hsa05030: Amyotrophic lateral sclerosis (ALS) VS. GO:0008624∼ induction of apoptosis by extracellular signals	GSP	0	0.009	0.016153439
hsa05219: Bladder cancer VS. GO:0044444∼ cytoplasmic part	GSN	0	0	0.000597928
hsa04664: Fc epsilon RI signaling pathway VS. GO:0046456∼ icosanoid biosynthetic process	GSN	0.001	0	0.001236482
hsa05040: Huntington's disease VS. GO:0030685∼ nucleolar preribosome	GSN	0	0.017	0.002211231
hsa04012: ErbB signaling pathway VS. GO:0050877∼ neurological system process	GSN	0	0	0.00252409
hsa00561: Glycerolipid metabolism VS. GO:0010033∼ response to organic substance	GSN	0.019	1	0.00258624
hsa04614: Renin-angiotensin system VS. GO:0004245∼ neprilysin activity	GSN	0	1	0.002828214
hsa04130: SNARE interactions in vesicular transport VS. GO:0006906∼ vesicle fusion	GSN	0.002	1	0.005413028
hsa00252: Alanine and aspartate metabolism VS. GO:0016885∼ ligase activity, forming carbon-carbon bonds	GSN	0	0.104	0.005649569
hsa05212: Pancreatic cancer VS. GO:0050801∼ ion homeostasis	GSN	0	0.007	0.008080875
hsa04350: TGF-beta signaling pathway VS. GO:0045687∼ positive regulation of glial cell differentiation	GSN	0	1	0.008538547

These GSP and GSN pairs are at the border of the Fisher exact test significance level p = 0.01. The full table is shown in Table S8 in File S2.

## Discussion

The improved performance of CoCiter over the Gene-Gene association analysis suggests that directly testing the gene-function association by defining functions in free terms could circumvent the limitations introduced by the insufficient gene annotations in either the GO or KEGG database. As traditional ways of analysing functional similarity between two gene sets are based on the significance of overlap between the two gene sets, they are unable to identify the same functions between the two gene sets if they have no overlapping genes, while CoCiter is able to find such hidden functional relationships, without requiring explicit gene overlap, by directly examining co-citations between the genes and functions.

It should be noted that although many co-citation tools exist, so far none of them aims to or is able to test the function enrichment of a gene set without predefined terms (see the comparisons of these tools with CoCiter in Table 1 and Results). This makes CoCiter a unique text-mining alternative to GO/KEGG-based functional enrichment analysis. Moreover, although tools like GSEA [24] and PAGE [25] are frequently used to test the significance of function/pathway enrichment, they require a ranked list, which is unavailable for either the query or target gene/term set.

The bottleneck of text mining-based tools is the time-consuming step of querying against the vast literature base. We implemented many optimizations to speed up the query. As a result, CoCiter search is already magnitudes faster than other text mining based tools (see Table 2/3 for the detailed statistics). Our test results show “A 1000×1000 Gene-Gene query with 1000 permutations takes ∼200 seconds on average”, “A 1000×16 Gene-Term query with 1000 permutations takes ∼270 seconds on average”. The detailed performance test of the CoCiter Gene-Gene function and the Gene-Term function are also shown in Figure S2/S3 (Supplemental Notes in File S1).

However, in rare cases a query can indeed take more time, for example, for some star genes and terms, such as “TP53” and “cancer”. Thus, CoCiter provides three utilities to solve the waiting time issue. The first and easiest way is users could provide their email address to CoCiter, and CoCiter will then automatically send the results to the email when the query finished; Second, CoCiter provides a unique job ID for each query, which could be used to check the query status or retrieve the query results at any time; Third, by using the SOAP API that CoCiter provides, the results can be automatically retrieved and stored in local files when the query finished.

Alternative studies with mechanistic/network based approaches have been used to infer the relationships between two gene sets [10]–[12]. However, none of these tools could be used to infer relationships between one gene set to any free term, which is the key feature of CoCiter. CoCiter also avoids many difficulties encountered when calculating functional similarity using standard GO terms [26], [27] (Supplemental Methods in File S1, Figure S4), because it directly links genes to user-defined genes/terms by comparing the observed co-citation frequency to the random expectation.

Three recently published studies [28]–[30] have already used CoCiter to infer relationships of a set of genes to specific terms (such as aging and various stresses) or to evaluate the biological coherency of genes within a small molecular network. These studies in addition to the tests and analyses provided here proved CoCiter as an extremely useful, flexible and convenient tool.

## Supporting Information

Figure S1 **The distribution of the co-citation paper counts of genes in the original manually curated “gene2pubmed” table.**
(EPS)

Figure S2 **Performance test of CoCiter Gene-Gene function (1000 permutations) with gene sets of the same size, which are randomly sampled from all human Entrez genes.**
(EPS)

Figure S3 **Performance test of CoCiter Gene-Term function (1000 permutations) with gene sets of the same size (randomly sampled from all human Entrez genes) and term sets (randomly sampled from a predefined biological term set).**
(EPS)

Figure S4 **The ∼18000 validated human PPIs in STRING are sorted to 5000 gene bins according to their confidence scores and the average CIs are plotted against the average confidence scores in each bin.**
(EPS)

File S1 **Supplemental Material.** The Supplemental Material includes Supplemental Methods, Supplemental Notes, and Supplemental Tables.
(DOC)

File S2 **Tables** **S3–S8.** This file includes Table S3, Table S4, Table S5, Table S6, Table S7, Table S8.
(XLS)
