# Supplementary material for: CoCiter: An Efficient Tool to Infer Gene Function by Assessing the Significance of Literature Co-Citation
Source: PLoS One. 2013 Sep 23;8(9):e74074. doi: 10.1371/journal.pone.0074074 (PMC3781068; doi:10.1371/journal.pone.0074074)
Supplement: File S1 — Supplemental Material. The Supplemental Material includes Supplemental Methods, Supplemental Notes, and Supplemental Tables. (DOC) [file pone.0074074.s005.doc]

# Supplemental Material for: “CoCiter: an efficient tool to infer gene function by assessing the significance of literature co-citation”

**Nan Qiao ^123#^, Yi Huang ^13#^ , Hammad Naveed^1^, Christopher D. Green^1^, and Jing-Dong J. Han^1*^**

**^1^**Chinese Academy of Sciences Key Laboratory of Computational Biology, Chinese Academy of Sciences-Max Planck Partner Institute for Computational Biology, Shanghai Institutes for Biological Sciences, Chinese Academy of Sciences, Shanghai, China, ^2^Center of Molecular Systems Biology, Institute of Genetics and Developmental Biology, Chinese Academy of Sciences, Beijing, China, **^3^**University of Chinese Academy of Sciences, Beijing, China.

Content

[Supplemental Material for: “CoCiter: an efficient tool to infer gene function by assessing the significance of literature co-citation” 1](#__RefHeading___Toc363767904)

[Supplemental Methods 3](#__RefHeading___Toc363767905)

[Curation of the GSP/GSN datasets 3](#__RefHeading___Toc363767906)

[Evaluating functional similarities between genes 4](#__RefHeading___Toc363767907)

[Evaluating the "genename + gene" rule 4](#__RefHeading___Toc363767908)

[Supplemental Notes 5](#__RefHeading___Toc363767909)

[Star Papers 5](#__RefHeading___Toc363767910)

[Speed Test 5](#__RefHeading___Toc363767911)

[Supplemental Tables 7](#__RefHeading___Toc363767912)

[Table S1. Evaluation of the "genename + gene" rule by manually examining 50 randomly selected human genes against the PubMed abstracts and full papers. 7](#__RefHeading___Toc363767913)

[Table S2. The 16 common organisms whose PubMed abstracts were indexed by Lucene. 9](#__RefHeading___Toc363767914)

[Table S3. The disease resistance genes and a randomly selected gene set (Entrez gene IDs). 9](#__RefHeading___Toc363767915)

[Table S4. The plasma membrane protein coding genes and nuclear protein coding genes (Entrez gene IDs). 9](#__RefHeading___Toc363767916)

[Table S5. The GSP dataset. 10](#__RefHeading___Toc363767917)

[Table S6. The GSN dataset. 10](#__RefHeading___Toc363767918)

[Table S7. Terms that are ignored in the Term Set input. Adapted from the Top 5000 list at http://www.wordfrequency.info on Jul 18, 2012. 10](#__RefHeading___Toc363767919)

[Table S8. Significance of association for the GSP and GSN gene and term sets detected by CoCiter or the GO-based Fisher exact test analysis. 10](#__RefHeading___Toc363767920)

## Supplemental Methods

### Curation of the GSP/GSN datasets

To create the GSP and GSN datasets for testing the accuracy and coverage of CoCiter, two independent gene-annotation databases GO and KEGG are used (downloaded from DAVID Knowledgebase in Jan, 2011). The DAVID KEGG dataset contains 201 gene sets, each of which has one KEGG pathway name to describe the pathway to which the gene set belongs. The DAVID GO dataset contains 8989 gene sets, each of which has one GO term to describe the function of the gene set.

The GSP dataset is defined as pairs of KEGG and GO gene sets sharing similar functions, such as the KEGG gene set “hsa00272:Cysteine metabolism” and GO gene set “GO:0006534~cysteine metabolic process”. The GSN dataset is defined as pairs of KEGG and GO gene sets sharing distinct and unrelated functions, such as the KEGG gene set “hsa00380:Tryptophan metabolism” and GO gene set “GO:0005795~Golgi stack”. As the KEGG pathway names are more concise than GO terms, we use the reduced KEGG pathway names (such as the keyword “Cysteine” for KEGG pathway “Cysteine metabolism”, the keyword “Terpenoid” for KEGG pathway “Terpenoid biosynthesis”, Table S5, 6) as the keywords to find the GO terms that have similar or distinct functions. When using these keywords as patterns to search the GO terms, the exactly matched GO terms together with the KEGG pathway are regarded as pairs of gene sets with similar function (GSP). Alternatively, three unmatched GO terms together with this KEGG pathway are defined as pairs of gene sets with distinct functions (GSN). In the end, 2097 GSP KEGG-GO pairs (Table S5) and 603 GSN KEGG-GO pairs are collected (Table S6).

### Evaluating functional similarities between genes

To test if CI could be used to evaluate the quality of new experimentally derived PPIs, we sorted the ~18000 validated human PPIs in the STRING database into 5000 gene bins according to their confidence scores and plotted the average CI versus the average confidence score in each bin. This result was compared with 5000 randomly selected protein pairs, each containing one protein located in the nucleus and the other located in the plasma membrane as these pairs are unlikely to have physical interactions. We find that all bins of validated STRING PPIs have significantly higher CIs than the sampled nucleus-plasma membrane protein pairs (*t* test p values < 2.2e-16), while higher CIs are observed among high confidence PPIs (Figure S4).

### Evaluating the "genename + gene" rule

The "genename + gene" rule was used to expand the original “gene2pubmed” dataset. To evaluate the accuracy of the "genename + gene" rule, we randomly selected 50 human genes and searched the phrase "genename + gene" in PubMed. Seventeen genes had hits in their search results, and by manually checking the first hit of these seventeen, we found that all of the gene names exist in the abstracts or the main text (Table S1).

## Supplemental Notes

### Star Papers

Some papers are linked to a large number of genes. However, it may be inappropriate to ignore them by simply removing them. We have solved this problem by using the permutation approach. If one paper is linked to a large number of genes, it will be more frequently hit in the permutation process, thus it will not affect the p value too much. Assuming a paper covers ten percent of all the genes, when randomly sampling a gene one thousand times, the chances that the gene is linked with the paper is 0.1. Therefore, the final p value will be around 0.1, which means that the influence of the paper on the co-citation significance of the gene will not be significant. As a result, papers that cover more than 5% of all genes, which can be regarded as star papers, will be determined as insignificantly linked to a gene by our methodology. This illustrates a major advantage of CoCiter over the other literature mining approaches. On the display page, a list of star papers, defined as the papers that include more than 100 genes, are listed separately at the bottom of the paper list.

### Speed Test

To test the speed of the CoCiter Gene-Gene function (1000 permutations), we randomly selected human gene set pairs with 6 different sizes (3, 10, 30, 100, 300 or 1000). For each pair size, we tried 10 queries of different sets of the same size, and calculated the average waiting time as a metric for speed. Most queries could finish within 1 minute, and a 1000x1000 Gene-Gene query with 1000 permutations takes ~200 seconds on average (Figure S2).

Similarly, to test the speed of theCoCiter Gene-Term function (1000 permutations), we randomly selected human gene sets with 6 different sizes (3, 10, 30, 100, 300 or 1000) and term sets with 4 different sizes (2, 4, 8, 16). For each pair size, we tried 10 queries of different sets of the same size, and calculated the average waiting time as a metric for speed. Most queries with a small number of terms could finish within 1 minute, and a 1000x16 Gene-Term query with 1000 permutations takes ~270 seconds on average (Figure S3).

We did observe that, for some star genes and terms, such as “TP53” and “cancer” (which is extreme case), it could take a longer time than expected. Nonetheless, using the CoCiter job ID the user can access their results once the job finishes. Moreover, if the user provides an email address in the query, the results are emailed to the user.

To avoid some frequent non-scientific terms and guarantee fast and accurate results, we have adapted a list (articles, conjunctions, demonstratives, prepositions, pronouns, interjections and several verbs, Table S7) from the top 5000 frequently-used words (Jul 2012, [http://www.wordfrequency.info](http://www.wordfrequency.info/)) to filter these words out during use of the Gene-Term function.

## Supplemental Tables

(Table S3-S8 are provided in a separate file “Table S3-S8.xls” in different tabs).

### Table S1. Evaluation of the "genename + gene" rule by manually examining 50 randomly selected human genes against the PubMed abstracts and full papers.

| **Entrez Gene ID** | **Entrez Gene Symbol** | **Synonyms** | **Match Result**^*^ |
| --- | --- | --- | --- |
| 1268 | CNR1 | CANN6|CB-R|CB1|CB1A|CB1K5|CB1R|CNR | Correct |
| 64434 | NOM1 | C7orf3|FLJ16401|SGD1 | Correct |
| 6776 | STAT5A | MGF|STAT5 | Correct |
| 2202 | EFEMP1 | DHRD|DRAD|FBLN3|FBNL|FLJ35535|MGC111353| | Correct |
| 80834 | TAS1R2 | GPR71|T1R2|TR2 | Correct |
| 51107 | APH1A | 6530402N02Rik|APH-1|APH-1A|CGI-78 | Correct |
| 2805 | GOT1 | GIG18 | Correct |
| 6304 | SATB1 | - | Correct |
| 25828 | TXN2 | MT-TRX|MTRX|TRX2 | Correct |
| 406988 | MIR205 | MIRN205 | Correct |
| 23500 | DAAM2 | KIAA0381|MGC90515|dJ90A20A.1 | Correct |
| 6129 | RPL7 | L7|MGC117326|humL7-1 | Correct |
| 5232 | PGK2 | PGKB|PGKPS|dJ417L20.2 | Correct |
| 10809 | STARD10 | MGC14401|NY-CO-28|PCTP2|SDCCAG28 | Correct |
| 975 | CD81 | CVID6|S5.7|TAPA1|TSPAN28 | Correct |
| 11091 | WDR5 | BIG-3|SWD3 | Correct |
| 9582 | APOBEC3B | APOBEC1L|ARCD3|ARP4|DJ742C19.2|FLJ21201| | Correct |
| 554236 | DPY19L2P1 | - | Not found |
| 442659 | RPL23P7 | RPL23_5_797 | Not found |
| 693215 | MIR630 | MIRN630|hsa-mir-630 | Not found |
| 100506689 | LOC100506689 | - | Not found |
| 728114 | LOC728114 | - | Not found |
| 100422313 | LOC100422313 | - | Not found |
| 645357 | LOC645357 | - | Not found |
| 163933 | FAM43B | FLJ44952 | Not found |
| 544317 | AASTH1 | - | Not found |
| 391803 | KRT18P45 | - | Not found |
| 100423017 | MIR3139 | - | Not found |
| 401959 | LOC401959 | FLJ20202 | Not found |
| 51079 | NDUFA13 | B16.6|CDA016|FLJ58045|FLJ59191|GRIM-19|G | Not found |
| 100271139 | RPL36AP9 | RPL36A_1_38 | Not found |
| 54532 | USP53 | DKFZp781E1417 | Not found |
| 100420189 | SEPHS1P3 | - | Not found |
| 150478 | bA395L14.12 | - | Not found |
| 100506622 | LOC100506622 | - | Not found |
| 100652761 | LOC100652761 | - | Not found |
| 400968 | LOC400968 | - | Not found |
| 284912 | LOC284912 | MGC3170 | Not found |
| 641382 | RP11-320L24.1-001 | - | Not found |
| 284365 | MGC45922 | - | Not found |
| 100189282 | TRNAK26 | - | Not found |
| 100421498 | MARK2P8 | - | Not found |
| 645895 | LOC645895 | - | Not found |
| 100288806 | LOC100288806 | - | Not found |
| 400206 | STELLAR | - | Not found |
| 56160 | NDNL2 | HCA4|MAGEG1|MAGEL3|NSE3|NSMCE3 | Not found |
| 653125 | LOC653125 | - | Not found |
| 171157 | SHFM5 | - | Not found |
| 359805 | PCNAP2 | pF2PCNA | Not found |
| 27095 | TRAPPC3 | BET3 | Not found |

^*^: These 50 randomly selected genes and their associated relationships were not used to evaluate the over 38 million relationships nor its accuracy, but were used to examine whether the search rule (Entrez gene name + word “gene”) can indeed identify the gene in the titles or abstracts.

### Table S2. The 16 common organisms whose PubMed abstracts were indexed by Lucene.

| **TaxID** | **Species** |
| --- | --- |
| 7165 | Anopheles gambiae |
| 3702 | Arabidopsis thaliana |
| 9913 | Bos taurus |
| 6239 | Caenorhabditis elegans |
| 9615 | Canis lupus familiaris |
| 7955 | Danio rerio |
| 7227 | Drosophila melanogaster |
| 9031 | Gallus gallus |
| 9606 | Homo sapiens |
| 28985 | Kluyveromyces lactis |
| 10090 | Mus musculus |
| 5141 | Neurospora crassa |
| 9598 | Pan troglodytes |
| 5833 | Plasmodium falciparum |
| 10116 | Rattus norvegicus |
| 4896 | Schizosaccharomyces pombe |

### Table S3. The disease resistance genes and a randomly selected gene set (Entrez gene IDs).

Provided as a separate spread sheet.

### Table S4. The plasma membrane protein coding genes and nuclear protein coding genes (Entrez gene IDs).

Provided as a separate spread sheet.

### Table S5. The GSP dataset.

Provided as a separate spread sheet.

### Table S6. The GSN dataset.

Provided as a separate spread sheet.

### Table S7. Terms that are ignored in the Term Set input. Adapted from the Top 5000 list at [**http://www.wordfrequency.info**](http://www.wordfrequency.info/) on Jul 18, 2012.

Provided as a separate spread sheet.

### Table S8. Significance of association for the GSP and GSN gene and term sets detected by CoCiter or the GO-based Fisher exact test analysis.

Provided as a separate spread sheet.
